# EOG Artifact Correction from EEG Recording Using Stationary Subspace Analysis and Empirical Mode Decomposition

**DOI:** 10.3390/s131114839

**Published:** 2013-11-01

**Authors:** Hong Zeng, Aiguo Song, Ruqiang Yan, Hongyun Qin

**Affiliations:** 1 School of Instrument Science and Engineering, Southeast University, Nanjing 210096, China; E-Mails: a.g.song@seu.edu.cn (A.S.); ruqiang@seu.edu.cn (R.Y.); 2 Department of Rehabilitation Medicine, Nanjing Tongren Hospital, Nanjing 211102, China; E-Mail: qinhy@njtrh.org

**Keywords:** electroencephalographic (EEG) signals, electro-oculographic (EOG) artifact, stationary subspace analysis (SSA), empirical model decomposition (EMD), signal reconstruction, artifact correction

## Abstract

Ocular contamination of EEG data is an important and very common problem in the diagnosis of neurobiological events. An effective approach is proposed in this paper to remove ocular artifacts from the raw EEG recording. First, it conducts the blind source separation on the raw EEG recording by the stationary subspace analysis, which can concentrate artifacts in fewer components than the representative blind source separation methods. Next, to recover the neural information that has leaked into the artifactual components, the adaptive signal decomposition technique EMD is applied to denoise the components. Finally, the artifact-only components are projected back to be subtracted from EEG signals to get the clean EEG data. The experimental results on both the artificially contaminated EEG data and publicly available real EEG data have demonstrated the effectiveness of the proposed method, in particular for the cases where limited number of electrodes are used for the recording, as well as when the artifact contaminated signal is highly non-stationary and the underlying sources cannot be assumed to be independent or uncorrelated.

## 1. Introduction

Quantitative analysis and interpretation of human electroencephalographic (EEG) signals can benefit various applications, such as the study of human brain functional states, to evaluate drug effects, to diagnose psychiatric and neurological disorders, to use brain-controlled devices to assist disabled people through Brain-Computer Interfaces (BCIs) and so on.

EEG signals are recorded from the scalp surface around the head with electrodes, which, however, may be contaminated by interferences. Eye movements and blinks constitute a major source of artifacts in EEG recordings, and such artifacts are commonly referred to as ocular artifacts (OA) or electrooculographic (EOG) artifacts [1]. In general, the EOG artifacts are characterized by localized patterns with higher amplitude and lower frequency than those of the EEG signals. Their vertical projection propagates quite symmetrically in an anterior-posterior direction. The potentials generated by ocular activity interfere with the electric field of neural origin mainly in the anterior scalp regions [2]. Thereby, a critical point in EEG signal processing is the need for careful treatment and reduction of these artifacts, which contaminate the EEG recordings and can lead to incorrect results and conclusions.

A classic way to deal with EOG artifacts is to instruct the person to avoid eye movements and blinks. This solution, however, may suppress and eventually harm the person's ongoing cognitive processes, thereby affecting the neuroscientific interpretation of the results [3–6]. Moreover, such a method is sometimes infeasible, especially in experiments performed on children, disabled people, uncooperative subjects, *etc.* [7,8]. Another early common practice is to simply reject the epochs contaminated with artifacts. However, not only the task-relevant neural responses are thrown away by such an approach, but also the scarcity of data or a high percentage of contamination makes this method unusable in many neural studies [9,10].

A practical solution for dealing with EOG artifacts is the procedure of artifact correction, where the artifact-laden epochs are “cleaned” by eliminating the artifacts and thus the cerebral activity is recovered. A variety of methods have been developed to this end, which can be generally categorized into two classes. One class is based on regression in the time domain, in which a proportion of the EOG recording is “subtracted” from each scalp electrode [11–13]. Such a method is quite simple, nevertheless, its performance is seriously affected by the well-known bidirectional contamination problem, which is due to the fact that the EOG signals also capture underlying neural activity originating from the prefrontal cortex, so the EOG and EEG recordings in the prefrontal region have common cerebral patterns, leading to their removal by regression scheme [3]. The second class of EOG artifact correction methods is based on the blind source separation (BSS) techniques. The BSS methods are capable of separating a mixture of signals originating from different sources to several components. Once the artifactual components are identified, they can be removed during the inverse BSS transformation to yield the clean cerebral activity data. Currently, such BSS-based methods have been shown to be very useful [14–17]. However, there are also several main problems of this methodology.

First, the classic BSS techniques such as independent component analysis (ICA), second-order blind identification (SOBI), may not be effective on the highly non-stationary EOG artifact-contaminated EEG recordings. On one hand, the ocular artifacts resulting from eye movements and blinks demonstrate strongly non-stationary characteristics: they are often localized with abruptly large amplitude and low frequency; their duration and amplitude appear to differ stochastically and considerably between successive eye movements or blinks. This implies that there are significant distribution changes in the artifact-contaminated EEG observations, such as the changes in the mean and the covariance matrix. However, ICA is not devoted to the understanding of the distribution changes, but to find the components that are both statistically independent and non-Gaussian [18–20]. Although SOBI exploits the temporal changes in the covariance matrix of the observations by the joint diagonalization of several covariance matrices with different time delays, the changes in the mean of the observations have not been taken into account. On the other hand, the underlying sources associated with artifacts may not be assumed to be independent or uncorrelated among each other [21]. It is known that ICA and SOBI can perform well on the eye blink artifacts contaminated EEG signals [16,19]. This is because blink artifacts mainly involve the vertical movement, the sources corresponding to the vertical movement and horizontal movement of eyes thus can be assumed to be independent or uncorrelated. However, when both the vertical and horizontal eye movements are involved in the contamination to the EEG observations, the sources associated with such EOG artifacts cannot be assumed to be independent nor uncorrelated anymore, because the vertical and horizontal eye movements are often accompanied with each other, in particular for eye ball rolls. Nevertheless, ICA instead has imposed the requirement of source-wise independency, while SOBI is based on the assumption that the sources should be uncorrelated with each other. Thus on the highly non-stationary EOG artifact-contaminated EEG recordings, we find that ICA and SOBI often fail to concentrate the artifacts in a small number of components. Since it is usually assumed that the number of sources is no greater than the number of channels in the BSS literature, in cases where there are limited number of electrodes used for EEG recording (e.g., often used in sleep studies [22] and when subjects are neonates or young infants, due to the size of the head [23]), it may lead to the loss of information related to brain activity by rejecting those sources found by ICA or SOBI.

Second, the components selected for removal may also contain neural activity aside from pure artifacts, in particular when there are limited numbers of recording electrodes [24–27]. In this sense, the removal of the contaminated components, followed by the signal reconstruction may lead to distortions of the underlying cerebral activity [17,19]. In order to recover the cerebral activity that has leaked into the components, the wavelet enhanced ICA (wICA) in [11] further makes use of wavelet decomposition and thresholding for denoising of the demixed components. Nevertheless, the performance of wICA largely depends on the quality of the sources separated by ICA, which may not be effective on the highly non-stationary EOG artifact-contaminated EEG recordings. Furthermore, during the wavelet decomposition, the wavelet basis and decomposition level need to be manually set, which may yield spurious harmonics because of the non-stationary and nonlinear characteristic of the signals. The inappropriate wavelet decomposition results may subsequently lead to less effective denoising through thresholding, causing distortions of the reconstructed signal eventually.

To address the above two problems of BSS-based methods, we propose a novel approach that aims to improve the performance of the EOG artifact correction on raw EEG recordings with a limited number of channels. The method utilizes stationary subspace analysis (SSA) [28,29] in conjunction with empirical model decomposition (EMD) [30]. Unlike the classic blind source separation with ICA or SOBI, the adopted BSS algorithm SSA is explicitly tailored to the understanding of distribution changes [29]. The type of distribution changes that SSA detects are changes in both the mean and the covariance matrix. In addition, neither independency nor uncorrelation is required among the sources by SSA. Subsequently, artifacts can be concentrated in only a few components. To the best of our knowledge, SSA has not been applied to EEG signals for removing artifacts thus far, even though it has been shown to have interesting applications in robust motor imagery prediction for Brain-Computer Interfaces [28], WiFi localisation [30], geophysical data analysis [29], computer vision [31] and change-point detection [32]. To further reduce the distortions in the SSA-corrected EEG, the EMD is then used to decompose those artifactual components for recovering cerebral activities that have leaked to these components. It is a completely data-driven signal decomposition method, the basis of the decomposition is adaptively derived from the data [33]. EMD usually demonstrates superior performance on non-stationary data to other methods [34–37], such as Fourier or the wavelet transforms, in which the basis needs to be manually specified. Experiments on both artificially contaminated data and publicly available real EEG recordings have been conducted, and the results show that the proposed method can effectively improve the artifact correction on raw EEG recordings

## 2. Proposed Approach for EOG Artifacts Correction

**Figure 1.**
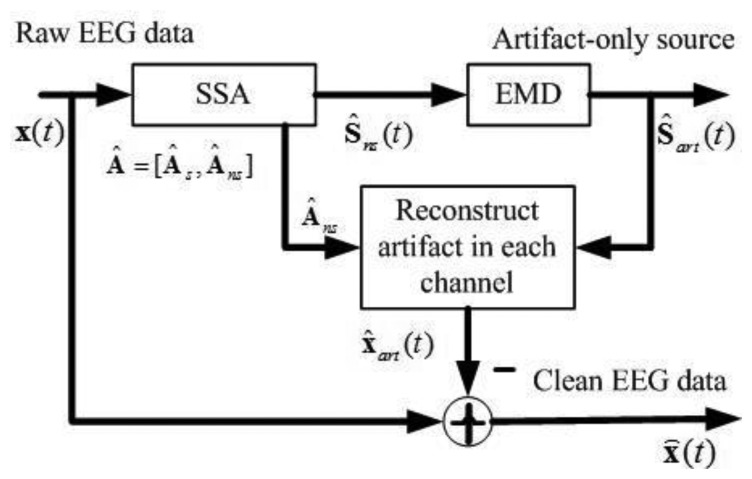
Block diagram of the proposed approach.

Figure 1 shows the block diagram of the proposed approach. It comprises the following key steps: (1) apply SSA to obtain the components containing artifacts **Ŝ***_ns_*(*t*) ; (2) apply EMD on such components to separate the artifact-only components **Ŝ***_art_*(*t*) and the cerebral activity that has leaked to **Ŝ***_ns_*(*t*); (3) project the artifact-only components back to each channel for estimating the artifacts, and then subtract the estimated artifacts **X̂***_art_*(*t*) from the raw EEG recordings to get a clean EEG.

### 2.1. Blind Source Separation by SSA

The first key step in the proposed approach is the application of SSA to separate the artifactual components from the raw EEG data. The observed signal **x**(*t*) is modeled as a linear superposition of two groups of sources with an invertible mixing matrix **A**. One group includes the sources **S***_s_*(t) related to the cerebral activity, the other are the sources **S***_ns_*(t) that arise from the eye movement and blink, *i.e.*, the highly non-stationary EOG artifactual sources whose distribution changes are the most pronounced:

(1) $$x(t)=AS(t)=\left[\begin{array}{cc} A_{s} & A_{ns} \end{array}\right]\;\left[\begin{array}{c} S_{s}(t)\\ S_{ns}(t) \end{array}\right]$$

where **A***_s_* and **A***_ns_* are the corresponding mixing matrices for **S***_s_*(t) and **S***_ns_*(t) respectively. To factorize the observed time series **x**(*t*) into the cerebral sources **S***_s_*(t) and the EOG artifactual sources **S***_ns_*(t), SSA is applied to estimate the inverse mixing matrix **A**^−1^ as **B**=[**B***_s_*^T^ **B***_ns_*^T^]^T^, such that **Ŝ***_s_*(*t*) = **B̂***_s_***x**(*t*) and **Ŝ***_ns_*(*t*) = **B̂***_ns_***x**(*t*) are the estimated cerebral and artifactual sources, respectively. The “weak non-stationarity” criterion is adopted in SSA, *i.e.*, the data are split into N consecutive epochs, and components are considered to be non-stationary if their empirical distribution (approximated by mean and covariance) changes significantly in these epochs [28,29]. It has been shown that such a criterion can be approximated as a generalized eigenvalue problem [29]:

(2) $$\begin{array}{ccccc} \underset{B}{\min} & \text{Tr}\left[BVB^{\text{T}}\right] & \text{subject} & \text{to} & B\bar{\Sigma }B^{\text{T}}=I \end{array}$$

with the matrix **V** defined by:

(3) $$V=\frac{1}{\text{N}}\sum_{k=1}^{\text{N}}\left\{\mu_{k}\mu_{k}^{\text{T}}+2\Sigma_{k}\bar{\Sigma }\Sigma_{k}^{\text{T}}\right\}-\bar{\mu }{\bar{\mu }}^{\text{T}}-2\bar{\Sigma }$$

where Tr[.] denotes the trace of a matrix, T is the transposition operation, **I** is the identity matrix, **μ***_k_* and **Σ***_k_* are the mean and covariance in the *k*^th^ epoch respectively, $\bar{\mu }=\frac{1}{\text{N}}\sum_{k=1}^{\text{N}}\mu_{k}$, $\bar{\Sigma }=\frac{1}{\text{N}}\sum_{k=1}^{\text{N}}\Sigma_{k}$. Such an objective function in Equation (3) essentially aims to minimize the distance between each epoch mean and covariance and their respective averages. This distance is measured using the variance of the mean and covariance across all epochs. This eigen-decomposition problem can be solved efficiently, and then the mixing matrix **Â** is obtained by **B̂**^−1^, the estimated components are derived by:

(4) $$\hat{S}(t)=\hat{B}x(t)$$

As can be observed from Equations (2) and (3), there are two advantages of SSA over ICA and SOBI. Firstly, it explicitly takes into account the distribution changes in both the mean and the covariance matrix. Secondly, it does not require the source-wise independency or uncorrelation. In fact, SSA allows arbitrary dependence structure among and between the two groups of sources [29]. Therefore, the highly non-stationary eye movement or blink artifacts generally will not spread out over multiple components but be concentrated into only a few components by SSA. Furthermore, SSA orders the components in increasing degree of non-stationarity [29], thereby the components that are likely to be associated with the artifacts would be ranked in the bottom. To automatically identify the artifactual sources **Ŝ***_ns_*(*t*), we adopted an effective method called ADJUST proposed in [38] recently.

### 2.2. EMD Denoising of Artifact Components

The identified artifactual components extracted by SSA are expected to correspond to artifacts only. However, when there are limited number of recording electrodes used, some neural activities usually have leaked to these artifactual components [11]. In other words, such components generally can be split into a high amplitude artifact **Ŝ***_art_*(*t*) localized in time with low frequency and a low amplitude cerebral signal **n̂**(*t*) with relatively high frequency:

(5) $${\hat{S}}_{ns}(t)={\hat{S}}_{\text{art}}(t)+\hat{n}(t)$$

The desired information may be lost if **Ŝ***_ns_*(*t*) is simply rejected during reconstruction. To retain the cerebral information, **Ŝ***_ns_*(*t*) needs to be denoised in order to obtain **Ŝ***_art_*(*t*), for which we propose to employ EMD.

The principle of EMD is to decompose a signal into a sum of the band-limited intrinsic mode functions (IMFs) by their characteristic time scales [30]. In specific, each IMF satisfies two basic conditions: (1) in the whole data set, the number of extrema and the number of zero crossings must be the same or differ at most by one; (2) at any point, the mean value of the envelope defined by the local maxima and the envelope defined by the local minima is zero. Here, the effective steps of EMD are briefly summarized as follows [30]:

(1) Given a single channel signal *S*(*t*), indentify all local maxima and minima.
(2) Interpolate between maxima to estimate the upper envelope *S_up_*(*t*) and between minima to estimate the lower envelope *S_low_*(*t*).
(3) Compute the mean of the two envelopes, *m*(*t*) = (*S_up_*(*t*) + *S_low_*(*t*))/2, and subtract it from the data: *d*(*t*) = *S*(*t*) − *m*(*t*).
(4) Repeat step (1)–(3) on *d*(*t*) until a stopping criterion is fulfilled.

The sifting process stops when the final residue *r*(*t*) is a constant, a monotonic function, or a function with only one maxima and one minima from which no more IMF can be derived. At the end of the decomposition, the signal *S*(*t*) can be represented as:

(6) $$S(t)=\sum_{i=1}^{\text{p}}d_{i}(t)+r(t)$$

where p is the total number of IMFs, *d_i_*(*t*)'s (*i* = 1,…,p) represent IMFs that are nearly orthogonal to each other and have zero mean. The first IMF owns the smallest time scale which corresponds to the fastest time variation of the signal. As the decomposition process proceeds, the time scale increases, and hence the mean frequency of the IMF decreases. In general, EMD is advantageous over other signal decomposition methods, such as the Fourier or the wavelet transforms, mainly because its basis of the decomposition is adaptively derived from the data, rather than manually set [30]. Moreover, EMD has demonstrated prominent performance on processing non-stationary signals, which is due to the fact that it is based on the local characteristic time scale of the data [19].

After applying EMD on each component in the artifactual group **Ŝ***_ns_*(*t*), the slowly varying trend representing the EOG artifact is expected to be captured by higher order IMFs (plus the final residue). We then separate **Ŝ***_art_*(*t*) from **Ŝ***_ns_*(*t*) by summing up the IMFs starting from *n*^th^ up to the residue, where the index *n* can be easily determined by visual inspection:

(7) $${\hat{S}}_{\text{art},j}(t)=\sum_{i=n}^{\text{p}}d_{i,j}(t)+r_{j}(t)$$

where **Ŝ***_art,j_*(*t*) denotes the *j*^th^ artifactual component, *d_i,j_* (*t*) represents the *i*^th^ IMF by applying EMD on the *j*^th^ artifactual component and *r_j_*(*t*) is the corresponding residue.

### 2.3. Reconstruct the EEG Signals

Using the estimated mixing matrix, **Â** = [**Â***_s_* **Â***_ns_*], given by SSA, these artifact-only components **Ŝ***_art_*(*t*) are projected back to EEG channel, and artifacts in EEG data **X̂***_art_*(*t*) are calculated as:

(8) $${\hat{x}}_{\text{art}}(t)={\hat{A}}_{ns}{\hat{S}}_{\text{art}}(t)$$

Finally, the clean EEG data **X̂**(*t*) is obtained by:

(9) $$\hat{x}(t)=x(t)-{\hat{x}}_{\text{art}}(t)$$

The complete steps of the proposed approach are summarized in Table 1.

## 3. Experimental Section

### 3.1. Suppression of Artifact on Artificially Contaminated EEG Signals

#### 3.1.1. Data Generation

**Figure 2.**
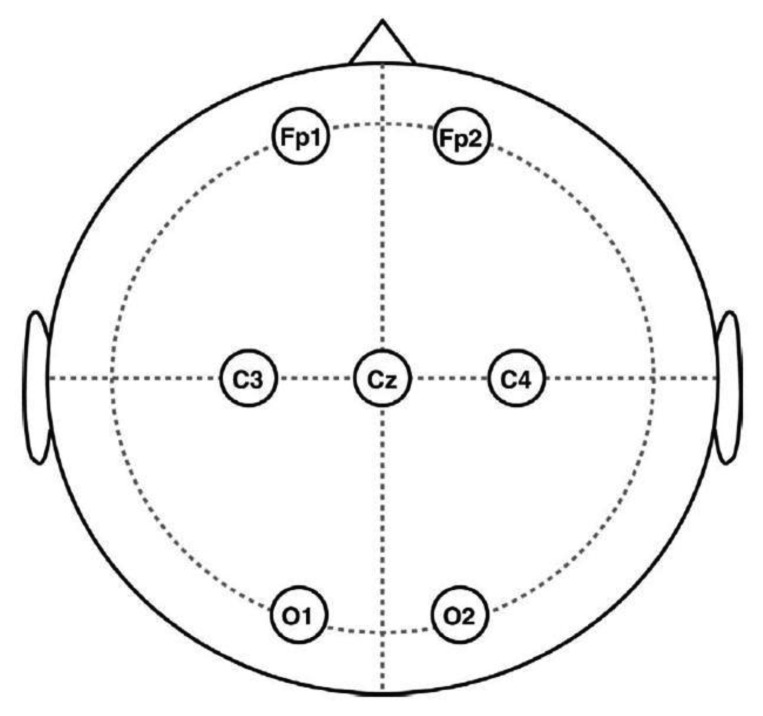
Placement of the EEG electrodes on the scalp according to the recording 10–20 system.

Forty healthy volunteers, 20 males and 20 females, aged between 20 and 33 years (mean age 27.6 years) were involved in the study. The EEG signals were recorded on 20 volunteers with the NeuroScan SynAmps2 system. Six EEG channels (Fp1, Fp2, C3, C4, O1, O2) were used for recording and the ground electrode was placed at position Cz, according to the 10–20 system (Figure 2). To obtain pure EEG data to be artificially contaminated, a 50-s consecutive epoch, where there were no obvious artifacts according to a careful inspection, was recorded for each volunteer in an eyes-closed session. Signals were digitized at a rate of 250 Hz, band-pass filtered at 0.5–50 Hz and notch filtered at 50 Hz.

To obtain the artifact signals for contaminating the pure EEG, separate EOG signals were obtained on the remaining 20 volunteers during eyes-open sessions with eye rolling, which were recorded by two electrodes placed above and below the left eye and another two on the outer canthi. This process gave rise to two bipolar signals for each volunteer, namely, vertical-EOG (VEOG), which is equal to the upper minus lower EOG electrode recordings and horizontal-EOG (HEOG), which is equal to the left minus right EOG electrode recordings. These EOG signals were band-pass filtered between 0.5 and 15 Hz.

Finally, to generate the “artificially contaminated EEG signals” (used for evaluating the performance of approaches for EOG artifact correction), we have used the Elbert's contamination model [39]:

(10) $${\text{mixed}}_{j}=\text{in}{\text{EEG}}_{j}+\alpha_{j}\text{VEOG}+\beta_{j}\text{HEOG}$$

where mixed*_j_* is the artificially contaminated EEG signal on the *j*^th^ channel and in EEG*_j_* is the collected artifact-free EEG signal described above. Variables α*_j_*, β*_j_* denote the propagation weights for VEOG and HEOG, respectively, initialized according to [40]. The mixing procedure by means of Equation (10) was carried out 20 times in order to simulate 20 subjects or sets of mixed signals.

**Figure 3.**
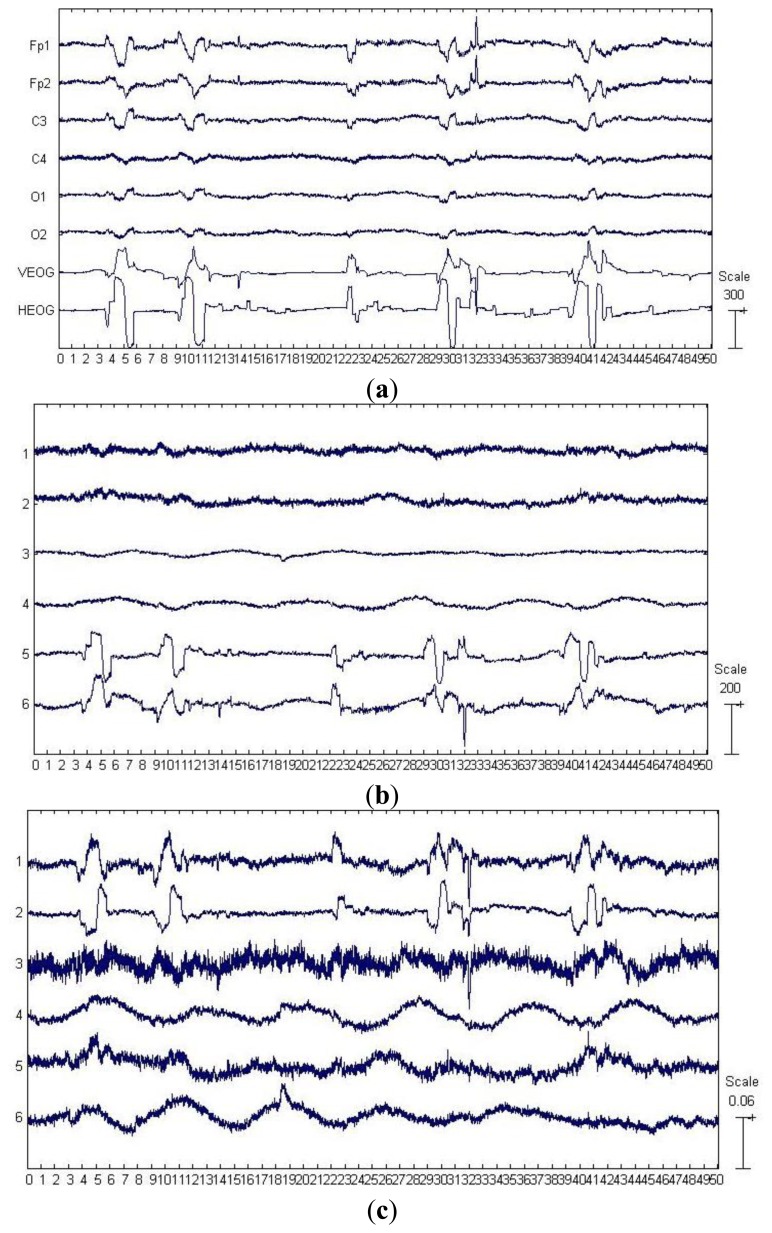

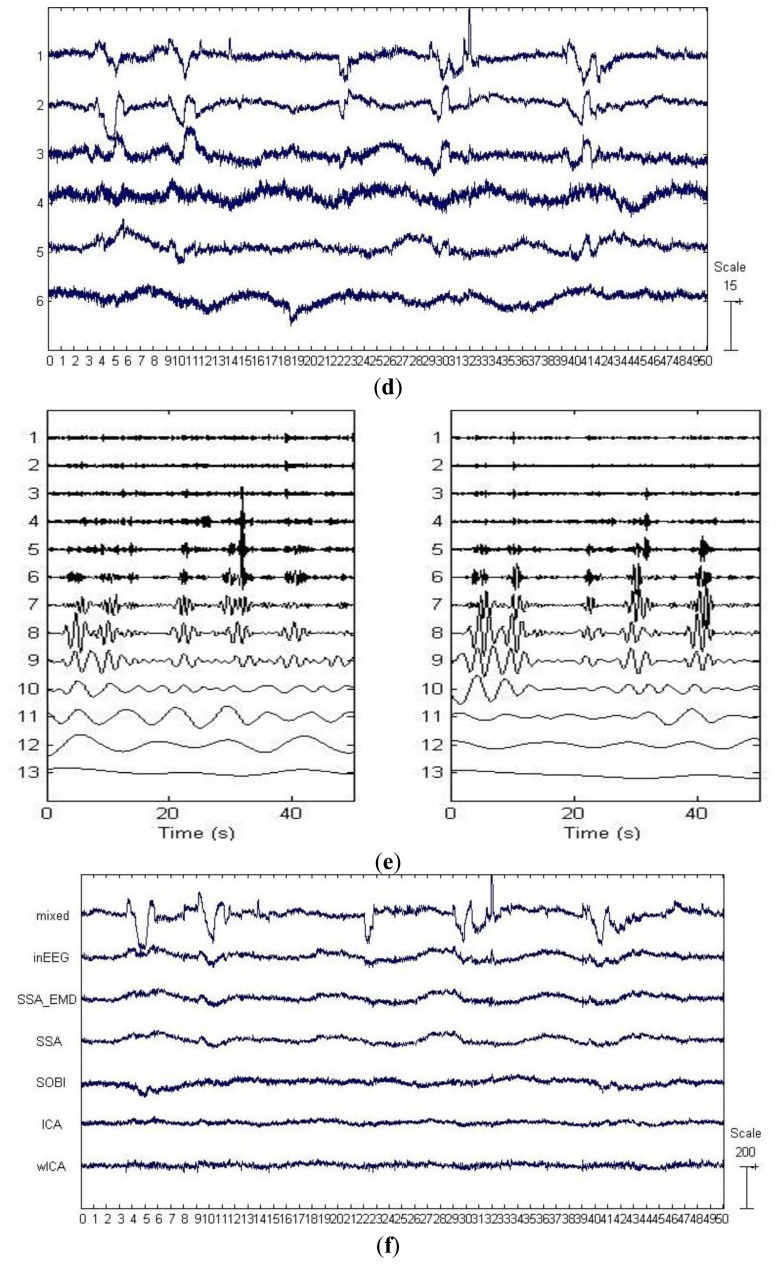

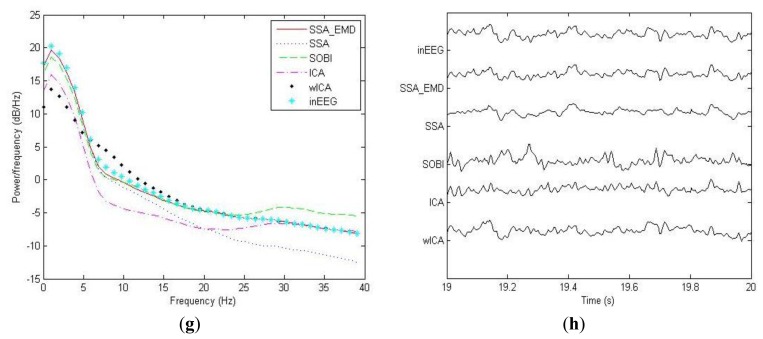
Results of EOG artifact removal on an example EEG data set from the 20 artificially contaminated ones. (**a**) The artificially contaminated EEG data with eye movement and blink artifacts (shown in the first six EEG channels) and the corresponding EOG signals used in the mixing procedure (shown in the last two channels); (**b**) Components separated by SSA; (**c**) Components separated by SOBI; (**d**) Components separated by ICA; (**e**) IMFs decomposed by applying EMD on the last two components in (b); (**f**) The mixed, the pre-contaminated EEG and the corrected EEG signals by each methods on channel Fp1; (**g**) The power spectrum estimates for the pre-contaminated EEG and the corrected EEG signals by each methods on channel Fp1; (**h**) The zoomed artifacts-corrected signals in the artifact-free epoch.

The first six channels in Figure 3a depict an example from one of the 20 artificially contaminated EEG signals, while the last two channels show the corresponding VEOG and HEOG used to generate the mixed signal. The eye movement and blink artifacts appear in EEG as big pulses localized in time and have a strong impact to EEG signals. Besides, the eye movement and blink episodes spread over all channels and affect most strongly the frontal sites (Fp1, Fp2).

#### 3.1.2. Performance Measures

Thanks to the pre-contaminated EEG signals described above, we were able to conduct quantitative comparison between the original and the corrected signals. Two commonly used evaluation metrics were adopted in the experiments with two goals: to test how successfully algorithms remove ocular artifacts, and how much the EEG signals are distorted after the artifact rejection procedure.

(1) Mutual Information
  The mutual information (MI) quantifies the mutual dependence of the pre-contaminated EEG signals and the artifact-corrected EEG data sets using the following formula [5]:
  (11) $$\text{MI}(\text{inEEG},\text{outEEG})=\sum_{x\in \text{inEEG}}\sum_{y\in \text{outEEG}}p(x,y)\log\left(\frac{p(x,y)}{p_{1}(x)p_{2}(y)}\right)$$
  where the inEEG denotes the artifact-free EEG data set before the contamination (Section 3.1.1) and the outEEG stands for the cleaned EEG signals by an approach for artifact correction. *p*(*x*,*y*) is the joint probability distribution function, and *p*_1_(*x*) and *p*_2_(*y*) are the marginal probability distribution functions of inEEG and outEEG, respectively. The larger the MI, the better the corrected EEG resembles the original EEG.
(2) Power Spectrum Distortion
  In order to quantify the spectral distortions across different frequency bands of the cerebral activity (Delta: 0.5–4 Hz, Theta: 4–8 Hz, Alpha: 8–12 Hz, Beta: 12–30 Hz and Gamma: 30–45 Hz), which were introduced by the artifact correction methods, the following index [5] was used:
  (12) $$\Delta P=\left|P_{\text{inEEG}}-P_{\text{outEEG}}\right|$$
  where *P*_inEEG_ and *P*_outEEG_ denote the power spectrum density estimated for the signals before and after applying an artifact correction approach, using the Welch method. The parameters used as input in the Welch algorithm are: 64 sample points as the window with 60 samples overlapped and 250 Hz sampling rate.

#### 3.1.3. Evaluation of Different Artifacts Correction Methods

The proposed method (referred to as SSA_EMD) was compared with SOBI-based approach (referred to as SOBI), ICA-based approach (referred to as ICA), SSA-based approach without EMD denoising (referred to as SSA) and wICA. For the compared approaches based only on SOBI, ICA or SSA, we first performed the blind source separation on the contaminated EEG signal, and then rejected the artifactual components automatically identified by the ADJUST algorithm (The implementation of ADJUST was downloaded from http://www.unicog.org/pm/pmwiki.php/MEG/RemovingArtifacts WithADJUST) during the reconstruction. For SOBI and ICA, we adopted the SOBI and infomax ICA algorithms implemented in the EEGLAB toolbox [41]. The implementation of wICA was obtained from authors of [11], and it is also based on infomax ICA from the EEGLAB toolbox. SSA was implemented based on the paper [29].

By applying SSA on the EEG data on the first six channels shown in Figure 3a, six components were obtained and depicted in Figure 3b. The last two components in Figure 3b were identified as the artifactual ones by ADJUST, which were strongly non-stationary, featuring abrupt pulses with large amplitude and short-duration. By observing the HEOG and VEOG signals that propagated onto the six EEG channels, we found that the last two artifactual components actually reflected the horizontal and vertical motion of eyes, respectively. Furthermore, these two artifactual components carried appreciable brain activity as well (see Figure 3b). Then EMD was applied on these two components for denoising them, and the resulting IMFs for each component are shown in two subfigures of Figure 3e. It can be observed that the relatively high oscillation both appears in the 1st IMF, the 2nd IMF and the 3rd IMF, which are likely to be associated with the cerebral signals. Thereby, the remaining IMFs of each artifactual component were summed together to reconstruct the artifact-only one. Finally, the artifact-only components were projected back to each channel for estimating the artifacts, and then the corrected EEG data were obtained by subtracting the estimated artifacts from the raw EEG recordings.

For the example data shown in Figure 3a, the components separated by SOBI and ICA are shown in Figure 3c,d, respectively. The ADJUST algorithm determined that both SOBI and ICA failed to capture the vertical and horizontal movements of eyes into two components, and the artifacts instead both spread into the first three components. wICA further performed the wavelet denosing on such a separation results by ICA for recovering the EEG.

#### 3.1.4. Evaluation Results

(1) Performance of removing the artifacts
  For the example EEG data set shown in Figure 3a, visual comparison of the mixed, the pre-contaminated EEG and the corrected EEG signals by different ocular artifacts removal methods is given in Figure 3f, where only the EEG on the Fp1 channel is depicted because the frontal channels near the eyes are contaminated most severely. The visual inspection confirms that all these five methods effectively suppress the EOG artifacts. However, it can be seen that compared to SOBI, ICA and wICA, both SSA and SSA_EMD give better approximations of the pre-contaminated EEG signals. Moreover, according to the mean and standard deviation of MI over 20 simulated EEG data sets which are reported in Table 2 (SSA:0.944 ± 0.105 *versus* SSA_EMD:1.036 ± 0.211), SSA_EMD outperforms SSA, indicating that SSA_EMD removes the ocular artifacts from the EEG signals most successfully among all the methods.
(2) Performance of distorting the cerebral activity
  For the example EEG data set shown in Figure 3a, we have shown the power spectrum density for the corrected EEG data by each method on the channel Fp1 in Figure 3g. It can be observed that ICA tends to underestimate the EEG power spectrum over all the frequency bands, except the range between 30 Hz and 40 Hz, indicating there is loss of brain activity with low frequency. This is confirmed by the zoomed ICA-corrected signal during an artifact-free epoch shown in Figure 3h, where the slow activities around 19.2 s, 19.4 s and 19.7 s in the pre-contaminated EEG signal have disappeared. SOBI has also considerably underestimated the power spectrum in the range of 0.5–4 Hz and eliminated the slow activities around 19.4 s and 19.9 s (see Figure 3h). It even overestimates the power spectrum density in the range of 25–40 Hz, which has led to spurious spiky behavior locally that does not present in the pre-contaminated EEG signal. Moreover, SSA seems to underestimate the EEG power spectrum in the range of 8–40 Hz, implying the neural activity of high frequency was undesirably cleaned. Accordingly, the zoomed plot of SSA-corrected signal in Figure 3h indicates that the fast oscillations in the original pre-contaminated EEG signal have vanished. For the wICA-corrected signal, there is a decrease in the power spectrum in the frequency range of 0.5–6 Hz as well as an increase in the frequency range of 6–20 Hz, indicating that wICA distorted the EEG signal considerably as can be seen in Figure 3h. By contrast, SSA-EMD has provided the best approximation of the power spectrum of the pre-contaminated EEG signal, as can be seen in Figure 3g. Table 2 shows the quantitative comparison results of Δ*P* for the proposed approach as well as other four methods over 20 simulated EEG data sets on channel Fp1. We can observe that SSA_EMD achieves the lowest errors of the power spectrum among all the approaches, indicating that EEG signals are less distorted by SSA_EMD than by the compared methods. The three spectral bands with the highest power spectrum errors in the EEG signal are the Delta, Theta and Alpha bands, where there may be the main incidence of ocular contamination in EEG channels.

#### 3.1.5. Discussion on the Evaluation Results

There are three important characteristics of the artificially contaminated EEG signals. One is that the EEG data set was recorded with limited number of channels (six channels, see Section 3.1.1), implying that the BSS algorithms can only separate the signal into six or even less underlying sources. Another is that the signal is highly non-stationary. The last is that the eye rolling artifactual sources used to construct the signal cannot be assumed to be independent or uncorrelated, since the vertical and horizontal movements of eyes were always accompanied with each other when eyes roll. On such a data set, both SOBI and ICA spread the artifacts in several components due to their drawbacks, considerable neural information thus got lost by rejecting more than necessary number of artifactual components during the reconstruction (see Figure 3g, Figure 3h and Table 2). In contrast, because the distribution changes in both the mean and the covariance matrix have been explicitly taken into account by SSA, and neither source-wise independency nor uncorrelation is assumed for the underlying sources, SSA succeeded to concentrate the EOG artifacts into fewer components. However, there is significant leakage of cerebral signals into them. Without applying EMD on these artifactual components, the residual cerebral activity cannot be recovered (see Figure 3g, Figure 3h and Table 2). Although wICA was also developed to utilize wavelet denosing for recovering the neural information, which has leaked into components by ICA, it worked on the separation results by ICA which has been found to be not very effective on such a kind of data, thus resulting in inferior performance to SSA_EMD.

### 3.2. Suppression of Artifact on the Real EEG Signals

#### 3.2.1. Data Description

**Figure 4.**
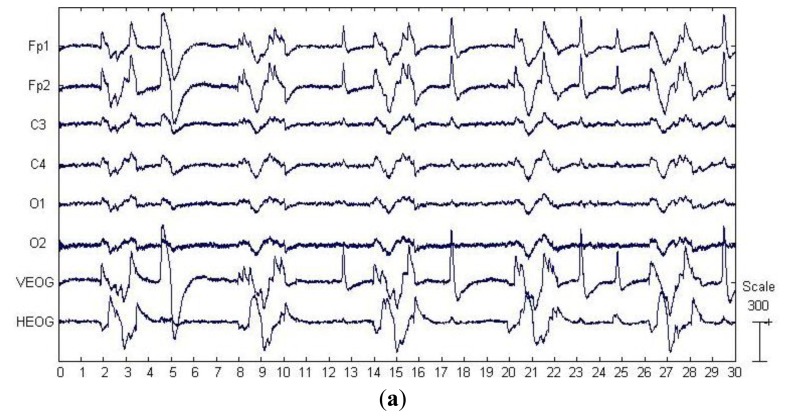

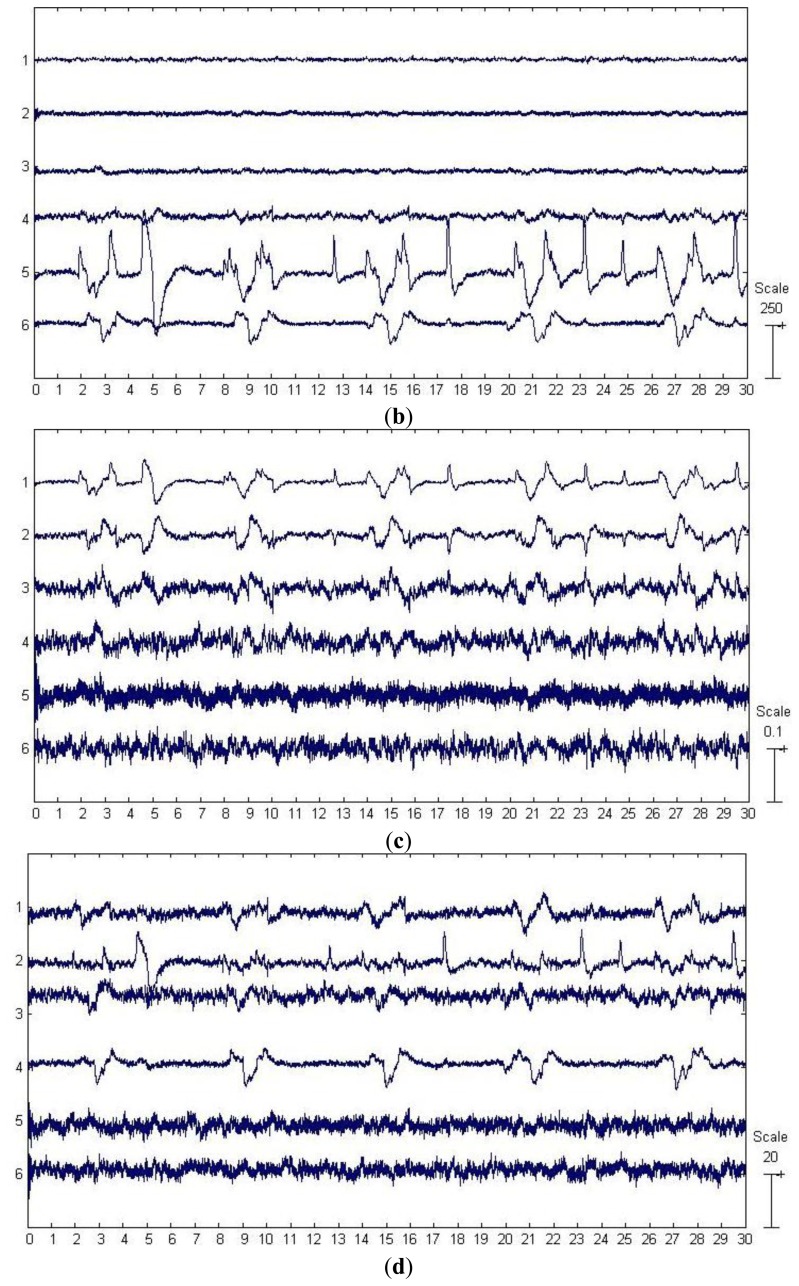

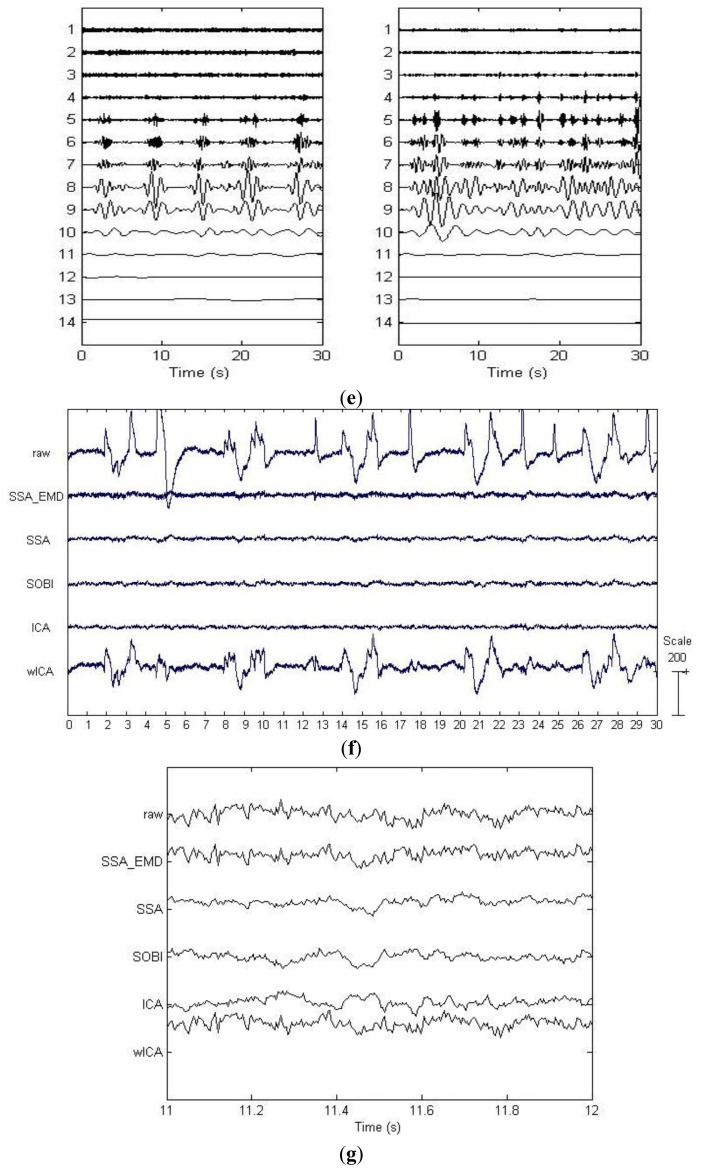
Results of EOG artifact removal on the real EEG data. (**a**) The raw EEG recordings with eye movement and blink artifacts, as well as the simultaneous recordings of the EOG signals; (**b**) Components separated by SSA; (**c**) Components separated by SOBI; (**d**) Components separated by ICA; (**e**) IMFs decomposed by applying EMD on the last two components in (b); (**f**) The raw EEG recording and the corrected EEG signals by each methods on channel Fp1; (**g**) The zoomed artifacts-corrected signals in the artifact-free epoch on channel Fp1.

In this section, we applied the artifact correction methods on a publicly available real EEG data set (downloaded from http://www.commsp.ee.ic.ac.uk/∼mandic/EEG-256Hz.zip). This data set also contains EOG artifacts from round movement of the eyes, which was recorded using the same collection configuration shown in Figure 2. In addition, the data set was sampled at 256 Hz and notch filtered at 50 Hz. As can be seen from the Figure 4a, which shows the real EEG signals, the EOG artifacts were present on all six EEG channels, while the artifacts are much stronger on the frontal lobe electrodes (Fp1, Fp2) and highly non-stationary (its amplitude differs between successive eye movements). Unlike the “artificially” contaminated EEG recordings, there is no ground truth “pure” EEG (pre-contaminated EEG), thus we can only present qualitative results in this section.

#### 3.2.2. Evaluation of Different Artifacts Correction Methods

For the proposed approach, the six components separated by SSA are shown in Figure 4b, where the last two components were identified as the artifactual ones by ADJUST. These two components accounted for the eye blinking and rolling. We have observed that these components carried some brain activities as well, which thus were further denoised by EMD. The resulting IMFs for each component are shown in two subfigures of Figure 4e. It can be observed that the relatively high oscillation both appears in the 1st–4th IMFs, which are likely to be associated with the cerebral signals. Thereby, the remaining IMFs of each artifactual component were summed together to reconstruct the artifact-only one. Finally, the artifact-only components were projected back to each channel for estimating the artifacts, and then the corrected EEG data were obtained by subtracting the estimated artifacts from the raw EEG recordings.

The results of components separation by SOBI and ICA are shown in Figure 4c,d, respectively. According to the results by ADJUST, SOBI grasped the time courses of the eye movement and blink artifacts in the first three components, while ICA located the artifacts in the top four components. wICA performed the wavelet denoising on all the six components by ICA before the EEG reconstruction.

#### 3.2.3. Evaluation Results

Figure 4f presents the visual comparison of the raw real EEG recording and the corrected EEG recording on Fp1 channel by different ocular artifacts correction methods. The EOG artifacts are effectively suppressed by all the five methods, except wICA, which has only suppressed the blink artifacts with a large amplitude and short-duration, but left over many eye movement artifacts in the corrected signals. Moreover, by observing zoomed signals during an artifact-free epoch in Figure 4g, we can find that SOBI, ICA and SSA have introduced considerable distortions, *i.e.*, the activities of high frequency in the original raw EEG signal have disappeared. In contrast, it is observed that SSA_EMD has effectively corrected the ocular artifacts without altering much of the high frequency cerebral activity.

#### 3.2.4. Discussion on the Evaluation Results

This real EEG recording has the same three characteristics as the artificially contaminated EEG signals. Both SOBI and ICA again failed to concentrate the artifacts in fewer components on this data set. Although SSA captured the eye blink and movements in only two components, such components also contained neural activity aside from the artifacts. Therefore, the removal of the contaminated components, followed by a signal reconstruction has led to distortions of the underlying cerebral activity (see Figure 4g). Nevertheless, by denoising the artifactual components found by SSA with the adaptive decomposition method EMD, the residual neural information was effectively recovered (see Figure 4g). Though wICA also attempted to recover the cerebral activity from the components by ICA, it only suppressed the blink artifacts with high amplitude (see Figure 4f) but still left over many eye movement artifacts. It is the manually set wavelet decomposition basis that might be blamed, which may only fit the eye blink artifacts well but may not match the eye movement ones with much more complicated localized temporal structures.

## 4. Discussion and Conclusions

Ocular artifacts represent a critical issue for quantitative EEG analysis, in particular when a limited number of channels are used for the recording, as well as when the contaminated signal is highly non-stationary and the underlying sources cannot be assumed to be independent or uncorrelated. In this paper, we have proposed an effective method for removing ocular artifacts from the raw EEG recording. It first performs blind source separation on the signal with SSA, which can concentrate the artifacts in only a few components. Then the adaptive signal decomposition technique EMD is applied on artifact sources to recover cerebral activity that has leaked to these sources. Experimental results on both the simulated data and real EEG recordings show that the proposed method not only effectively suppresses the highly non-stationary ocular artifacts, but also conserves much better the cerebral activity outside the artifact episodes.

The key to assure that the artifacts can be captured in fewer components so as to keep more neural information in the remaining cerebral sources is to choose a BSS algorithm with particularly designed strength, as well as proper underlying assumptions that most closely match the physical properties of the problem at hand. SSA's ability to separate highly non-stationary signals with arbitrary dependency among the underlying sources is one reason for the differences in the observed performance of SSA_EMD based approach versus the SOBI or ICA based ones. This is also the primary reason it was chosen for the proposed procedure on the eye movement (in particular eye roll) artifacts-contaminated EEG recordings with a limited number of channels. However, when there are enough electrodes for recording, such a problem is not so critical anymore. The discussion above is intended to enhance the awareness that BSS algorithms may not produce physical meaningful components unless they take the characteristics of the signals into account and their underlying assumption meets the properties of signals to be analyzed.

The rationality behind the combination of SSA and EMD will also be discussed. On one hand, although SSA is able to capture the artifacts in only a few components, there is still considerable neural activity carried by these artifactual components. Thereby it may lead to loss of cerebral information by simply rejecting the artifactual components during the reconstruction. On the other hand, despite the fact that EMD adaptively derives the decomposition basis from local dynamics in the data, it has been reported that EMD may fail to correct the artifacts by directly working on the raw EEG recordings [42]. This is because the ratio of artifact magnitude to the cerebral activity magnitude in a channel may not be high enough to separate them effectively [42].

Nevertheless, ocular artifacts can be concentrated in only a few components by SSA, where the ratio is much higher than in the artifact contaminated channel. Thus it is preferable to apply EMD on the artifactual components rather than directly on the raw recording. In other words, such a combination can substantially overcome the individual main drawbacks of SSA and EMD.

The main limitation of the proposed method is that the starting IMF for reconstructing the artifact-only component after applying EMD is identified manually. More efforts are needed in order to upgrade SSA_EMD to an automatic methodology. Besides, we have mainly focused on ocular artifacts and performance of other types of artifacts is yet to be investigated in the future work.

## Figures and Tables

**Table 1.** Summary of proposed approach for EOG artifacts correction from raw EEG recordings.

**Processing:**	
(1)	Apply SSA on **x**(*t*), and then identify the artifactual components **Ŝ***_ns_*(*t*) as well as the corresponding columns of mixing matrix **Â***_ns_*;
(2)	Perform EMD denoising for the artifactual components **Ŝ***_ns_*(*t*) to obtain artifact-only components **Ŝ***_art_*(*t*);
(3)	Estimate the artifacts in multichannel EEG data by **X̂***_art_*(*t*) = **Â***_ns_***Ŝ***_art_*(*t*);
(4)	Subtract the artifacts from EEG data to get clean EEG: **X̂**(*t*) = **X**(*t*) − **X̂***_art_*(*t*).

**Table 2.** MI and Δ*P* by different EOG artifact correction methods over 20 artificially contaminated EEG data sets on channel Fp1.

**Quantification Metrics**	**SOBI**	**ICA**	**wICA**	**SSA**	**SSA_EMD**
MI	0.102 ± 0.062	0.169 ± 0.084	0.059 ± 0.021	0.944 ± 0.105	**1.036 ± 0.211**
Δ*P* in Delta band (dB/Hz)	1.678 ± 2.227	4.317 ± 1.025	6.153 ± 2.59	0.674 ± 0.473	**0.669 ± 0.328**
Δ*P* in Theta band (dB/Hz)	1.739 ± 2.359	4.752 ± 2.468	1.927 ± 2.893	1.329 ± 1.564	**1.025 ± 0.946**
Δ*P* in Alpha band (dB/Hz)	0.684 ± 0.846	4.494 ± 3.156	1.586 ± 2.121	1.400 ± 1.791	**0.655 ± 0.269**
Δ*P* in Beta band (dB/Hz)	0.501 ± 0.297	2.176 ± 4.012	0.227 ± 0.319	2.835 ± 3.984	**0.147 ± 0.120**
Δ*P* in Gamma band (dB/Hz)	2.373 ± 4.682	0.118 ± 0.197	0.021 ± 0.147	4.060 ± 2.556	**0.006 ± 0.003**
